# Supplementary material for: SARS-CoV-2 D614G spike mutation increases entry efficiency with enhanced ACE2-binding affinity
Source: Nat Commun. 2021 Feb 8;12:848. doi: 10.1038/s41467-021-21118-2 (PMC7870668; doi:10.1038/s41467-021-21118-2)
Supplement: Supplementary file 1 — Supplementary Information [file 41467_2021_21118_MOESM1_ESM.pdf]

Supplementary information

SARS-CoV-2 D614G spike mutation increases entry efficiency with enhanced ACE2-binding affinity

Seiya Ozono, Yanzhao Zhang, Hirotaka Ode, Kaori Sano, Toong Seng Tan, Kazuo Imai, Kazuyasu Miyoshi, Satoshi Kishigami, Takamasa Ueno, Yasumasa Iwatani, Tadaki Suzuki, and Kenzo Tokunaga

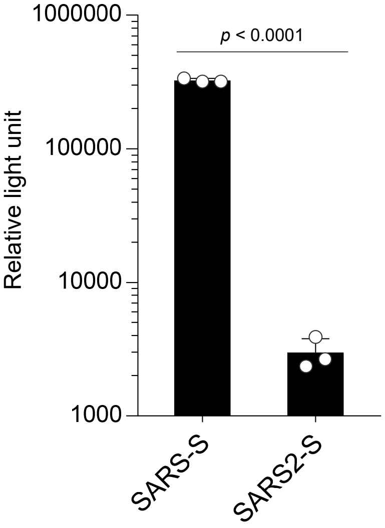

Supplementary Figure 1. ACE2 expression alone is insufficient to support SARS2-S-mediated cell entry. To prepare S-pseudotyped lentiviruses, 293T cells were transfected with the HiBiT-tagged lentiviral packaging plasmid, the firefly luciferase-reporter lentiviral transfer plasmid, and either a SARS-CoV S (SARS-S) or SARS-CoV-2 S (SARS2-S) expression plasmid. The viruses produced were assessed by HiBiT assays, and S-pseudotyped viruses normalized based on HiBiT activity were used for infection of 293T cells expressing the host receptor ACE2 only. Cell entry was determined by firefly luciferase activity in cell lysates. Data from three experiments are shown (mean ± s.d., n = 3 technical replicates). The p value was calculated using a two-tailed unpaired Student's t-test. Source data are provided as a Source Data file.

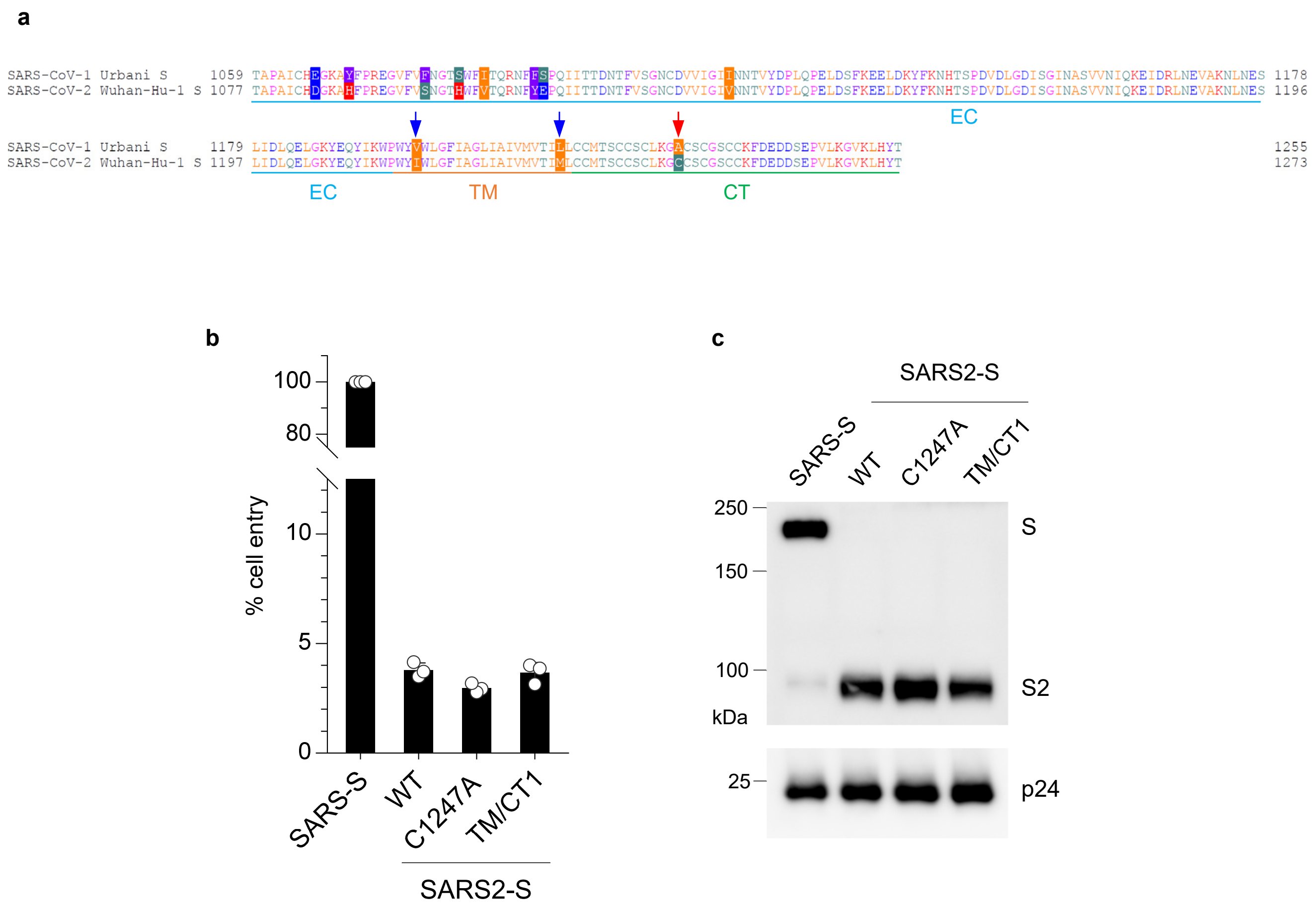

Supplementary Figure 2. The lower level of SARS2-S-mediated entry is not due to the incompatibility between SARS2-S and a lentiviral vector. (a) Amino acid sequence alignments of the C-terminal sequence of S proteins from SARS-CoV (Urbani strain) and SARS-CoV-2 (Wuhan-Hu-1 strain). Amino acid differences are boxed in several colors (polar neutral in green, aliphatic/hydrophobic in orange, aromatic/hydrophobic in purple, basic in red, and acidic in blue). EC, extracellular domain; TM, transmembrane domain; CT, cytoplasmic tail. SARS2-S-C1247A and SARS2-S-TM/CT1 were created by mutating a cysteine to an alanine at the CT (indicated by a red arrow) and by additionally mutating an isoleucine and a methionine to a valine and a leucine at the TM (indicated by blue arrows), respectively. (b) Cell entry of lentiviruses pseudotyped with SARS-S or SARS2-S (WT or mutants). 293T cells were transfected with the HiBiT-tagged lentiviral packaging plasmid, the firefly luciferase-reporter lentiviral transfer plasmid, and the plasmid expressing either the SARS2-S wild-type (WT) protein or one of the two mutants (SARS2-S-C1247A or SARS2-S-TM/CT1). Viruses produced were assessed by HiBiT assays, and S-pseudotyped viruses normalized based on HiBiT activity were used for infection of 293T cells expressing ACE2 and TMPRSS2. Cell entry was determined by firefly luciferase activity in cell lysates. Data from three experiments are shown as a percentage of cell entry of the SARS-S-pseudotyped viruses (mean ± s.d., n = 3 technical replicates). (c) Virion incorporation of SARS-S and SARS2-S proteins. Pelleted S-pseudotyped lentiviruses were subjected to immunoblot analyses using antibodies against p24 (lower) and T7 epitope tag to detect C-terminally tagged SARS-S or SARS2-S (upper). Data shown are representative of two independent experiments. Note that SARS2-S incorporated into virions is completely cleaved by host cell proteases, whereas that of SARS-S is uncleaved and results in a two-times larger band size, making it difficult to directly compare these S proteins. Source data for **b** and **C** are provided as a Source Data file.

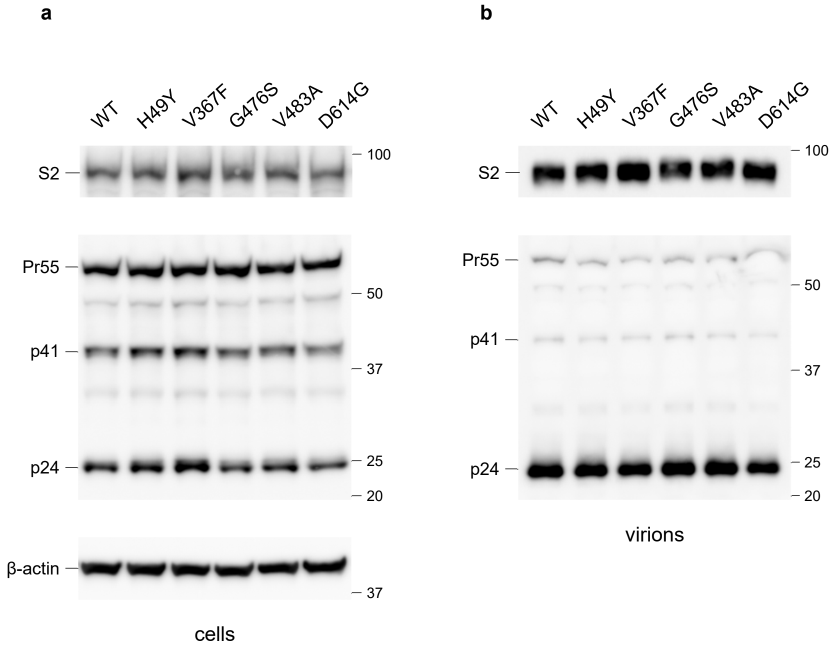

Supplementary Figure 3. Expression and virion incorporation of SARS2-S. Western blot analysis performed by using (a) lysates from transfected 293T cells or (b) purified lentiviral particles. Antibodies specific for S2 (upper), p24 (middle in *a*, lower in *b*), and β-actin (lower in *a*) were used. Data shown are representative of two independent experiments. Source data are provided as a Source Data file.

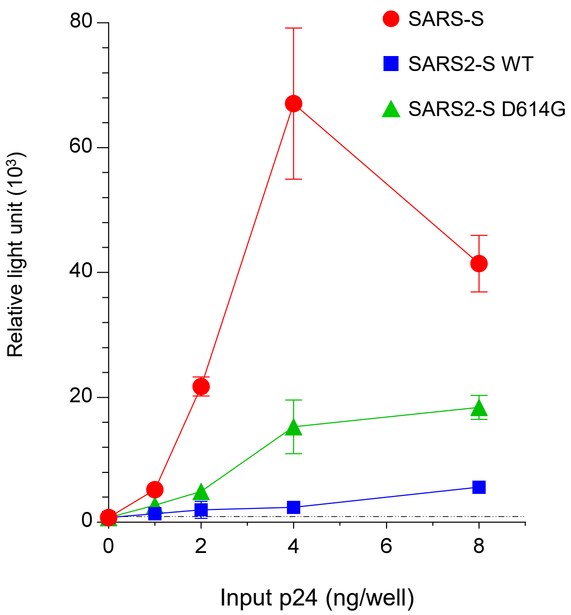

Supplementary Figure 4. Infection of human small airway epithelial cells by the S-pseudotyped viruses. The human small airway epithelial cells (2 x 10^4) were infected with increasing amounts of p24 antigen (1, 2, 4, and 8 ng) of lentiviruses pseudotyped with either SARS-S (red circle), SARS2-S WT (blue square), or SARS2-S D614G mutant (green triangle) SARS2-S. Cell entry was determined by luciferase assays. Representative data from two independent experiments are shown as relative light units (mean $\pm$ s.d., n = 2 technical replicates). The dashed line indicates the negative control background generated by spikeless lentiviruses. Source data are provided as a Source Data file.

		KD (M)	*Kon* ($M^{-1}S^{-1}$)	*Kdis* (S^{-1})
**WT**	**1st**	6.136E-10	1.58E+05	1.E-04
	**2nd**	8.761E-10	1.81E+05	2.E-04
	**3rd**	7.457E-10	1.75E+05	1.E-04
**D614G**	**1st**	2.97E-10	1.82E+05	5.E-05
	**2nd**	3.103E-10	1.76E+05	5.E-05
	**3rd**	2.31E-10	1.81E+05	4.18E-05
p value		0.0042	0.3076	0.0129

Supplementary Figure 5. Binding affinity between ACE2 dimer and either SARS2-S WT or D614G trimer. Shown are the association rate constants (*Kon*), dissociation rate constants (*Kdis*), and equilibrium dissociation constants (*KD* = *Kdis/Kon*) obtained from three independent experiments at 30°C using different protein preparations. The difference in *KD* was statistically significant by a two-tailed unpaired Student's *t*-test. Source data are provided as a Source Data file.

Supplementary Table 1: Primers used in this study

Designation	sequence
ACE2-Acc-S	GGGGTACCATGTCAAGCTCTTCCTGGCTC
ACE2-Xho-A	CCGCTCGAGCTAAAAGGAGGTCTGAACATC
TMPRSS2-BsiW-S	ATCGTACGCCATGGCTTTGAACTCAGGGTCAC
TMPRSS2-Xho-A	CCGCTCGAGTTAGCCGTCTGCCCTCATTTG
SARS-S-BsiW-S	ATCGTACGCCATGTTCATCTTCCTGCTGTTCCT
SARS-S-Xho-A	CCGCTCGAGTTAGGTGTAGTGCAGTTTCACTCC
SARS2-S-Acc-S	GGGGTACCATGTTTGTGTTCCTGGTGCT
SARS2-S-Not-A	ATTGGCGGCCGCTCTAGATCAGGTGTAGTGCAGTTTCAC
SARS/SARS2-S-Xho-A for C-tagging	CCGCTCGAGGGTGTAGTGCAGTTTCACTC
SARS2-S-H49Y-S	GGTGTTCAGGTCTTCTGTGCTGTACAGCACCCAGG
SARS2-S-H49Y-A	CCTGGGTGCTGTACAGCACAGAAGACCTGAACACC
SARS2-S-V367F-S	GGATTAGCAATTGTGTGGCTGACTACTCTTTCCTCTACAACTC
SARS2-S-V367F-A	GAGTTGTAGAGGAAAGAGTAGTCAGCCACACAATTGCTAATCC
SARS2-S-G476S-S	GATTTACCAGGCTAGCAGCACACCGTGTAATGGAGTGG
SARS2-S-G476S-A	CCACTCCATTACACGGTGTGCTGCTAGCCTGGTAAATC
SARS2-S-V483A-S	TGTAATGGAGCGGAGGGCTTCAATTGTTACTTTC
SARS2-S-V483A-A	GAAAGTAACAATTGAAGCCCTCCGCTCCATTACA
SARS2-S-D614G-S	CTCTACCAGGGTGTGAACTGCACTGAGGTG
SARS2-S-D614G-A	CACCTCAGTGCAGTTCACACCCTGGTAGAG
SARS2-S-C1247A-S	GTCTGAAAGGCGCCTGTTCCTGTGG
SARS2-S-C1247A-A	CCACAGGAACAGGCGCCTTTCAGAC
SARS2-S-TM/CT1-S	CAAGTGGCCATGGTACGTATGGCTGGGCTTCATCG
SARS2-S-TM/CT1-A	CGATGAAGCCCAGCCATACGTACCATGGCCACTTG
