# Supplementary material for: SARS-CoV-2 D614G spike mutation increases entry efficiency with enhanced ACE2-binding affinity
Source: Nat Commun. 2021 Feb 8;12:848. doi: 10.1038/s41467-021-21118-2 (PMC7870668; doi:10.1038/s41467-021-21118-2)
Supplement: Supplementary file 3 — Reporting Summary [file 41467_2021_21118_MOESM3_ESM.pdf]

nature research

Corresponding author(s): Kenzo Tokunaga

Last updated by author(s): Jan 7, 2021

Reporting Summary

Nature Research wishes to improve the reproducibility of the work that we publish. This form provides structure for consistency and transparency in reporting. For further information on Nature Research policies, see our Editorial Policies and the Editorial Policy Checklist.

Statistics

For all statistical analyses, confirm that the following items are present in the figure legend, table legend, main text, or Methods section.

n/a	Confirmed	
☐	☒	The exact sample size (*n*) for each experimental group/condition, given as a discrete number and unit of measurement
☐	☒	A statement on whether measurements were taken from distinct samples or whether the same sample was measured repeatedly
☐	☒	The statistical test(s) used AND whether they are one- or two-sided<br>*Only common tests should be described solely by name; describe more complex techniques in the Methods section.*
☒	☐	A description of all covariates tested
☒	☐	A description of any assumptions or corrections, such as tests of normality and adjustment for multiple comparisons
☐	☒	A full description of the statistical parameters including central tendency (e.g. means) or other basic estimates (e.g. regression coefficient) AND variation (e.g. standard deviation) or associated estimates of uncertainty (e.g. confidence intervals)
☐	☒	For null hypothesis testing, the test statistic (e.g. *F*, *t*, *r*) with confidence intervals, effect sizes, degrees of freedom and *P* value noted<br>*Give P values as exact values whenever suitable.*
☒	☐	For Bayesian analysis, information on the choice of priors and Markov chain Monte Carlo settings
☒	☐	For hierarchical and complex designs, identification of the appropriate level for tests and full reporting of outcomes
☒	☐	Estimates of effect sizes (e.g. Cohen's *d*, Pearson's *r*), indicating how they were calculated

Our web collection on statistics for biologists contains articles on many of the points above.

Software and code

Policy information about availability of computer code

Data collection	Raw luciferase data was exported to Microsoft Excel 2016 through MicroWin (v. 4.36.).<br>Western blot images were captured with Image Reader LAS-3000 (v.2.21).<br>Binding affinity was analyzed by Octet Data Analysis Software v11.1 (FortéBio).
Data analysis	Graphs were generated in GraphPad Prism version 8.04.<br>Structural figures were generated in PyMol (v.2.4.).<br>Sequence alignment was performed by Genetyx (v.13.1.2.).

For manuscripts utilizing custom algorithms or software that are central to the research but not yet described in published literature, software must be made available to editors and reviewers. We strongly encourage code deposition in a community repository (e.g. GitHub). See the Nature Research guidelines for submitting code & software for further information.

Data

Policy information about availability of data

All manuscripts must include a data availability statement. This statement should provide the following information, where applicable:

- Accession codes, unique identifiers, or web links for publicly available datasets
- A list of figures that have associated raw data
- A description of any restrictions on data availability

Source Data are provided with this paper. A complete list of all primers used in this study is provided in Supplementary Table 1. The templates used to create structural models are available from PBD code 6VYB (https://www.rcsb.org/structure/6VYB), 6M0J (https://www.rcsb.org/structure/6M0J), 6CS1 (https://www.rcsb.org/structure/6CS1), and 3D0G (https://www.rcsb.org/structure/3D0G). The GenBank reference sequences of SARS-S, SARS2-S and human ACE2 used for the modeling are available from GenBank IDs: NP_828851.1, YP_009724390.1, and NP_001358344.1, respectively. The codon-optimized SARS2-S sequence is

available from Sino Biological's website (https://www.sinobiological.com/cdna-clone/2019-ncov-cov-spike-vg40589-ut). All plasmids used in this study are available from the corresponding author under a material transfer agreement with the National Institute of Infectious Diseases.

Field-specific reporting

Please select the one below that is the best fit for your research. If you are not sure, read the appropriate sections before making your selection.

[x] Life sciences [ ] Behavioural & social sciences [ ] Ecological, evolutionary & environmental sciences

For a reference copy of the document with all sections, see nature.com/documents/nr-reporting-summary-flat.pdf

Life sciences study design

All studies must disclose on these points even when the disclosure is negative.

Sample size	No sample size calculation was performed. Sample sizes were determined based on the number of available information of natural variants and patient sera (at the timing of our initial study). All experiments were performed with at least two biological replicates.
Data exclusions	We did not exclude any data for the experiments presented.
Replication	All experiments for cell entry were performed with three technical replicates on at least three biological replicates. Neutralization assays were performed with three technical replicates on at least two biological replicates. Successful replication was confirmed.
Randomization	Randomization is not relevant to our study because this was not a clinical trial and there were no samples allocated into control and experimental groups.
Blinding	Blinding was not performed because this was not a population-based case-control study, and was not possible because these experiments were carried out using in vitro materials that we prepared by ourselves.

Reporting for specific materials, systems and methods

We require information from authors about some types of materials, experimental systems and methods used in many studies. Here, indicate whether each material, system or method listed is relevant to your study. If you are not sure if a list item applies to your research, read the appropriate section before selecting a response.

Materials & experimental systems

n/a	Involved in the study	
[ ]	[x]	Antibodies
[ ]	[x]	Eukaryotic cell lines
[x]	[ ]	Palaeontology and archaeology
[x]	[ ]	Animals and other organisms
[ ]	[x]	Human research participants
[x]	[ ]	Clinical data
[x]	[ ]	Dual use research of concern

Methods

n/a	Involved in the study	
[x]	[ ]	ChIP-seq
[x]	[ ]	Flow cytometry
[x]	[ ]	MRI-based neuroimaging

Antibodies

Antibodies used	Anti-SARS-CoV-2 Spike mouse monoclonal antibody (GeneTex, GTX632604, 1:1000)<br>Anti-T7 epitope mouse monoclonal antibody (Novagen, 69522-4, 1:3000)<br>Anti-p24 mouse monoclonal antibody (Nu24 made in-house, 1:1000)<br>Anti–β-actin mouse monoclonal antibody (Sigma-Aldrich, A5316, 1:5000)<br>Peroxidase-conjugated AffiniPure goat anti-mouse IgG (Jackson ImmunoResearch Laboratories, 115-035-062, 1:10000)
Validation	The anti-SARS-CoV-2 Spike mouse monoclonal antibody, which was generated against SARS-CoV SΔ10 protein (1029-1192 a.a.), detects S2 subunit of both SARS-S and SARS2-S proteins, and was validated by the manufacturer for WB and IF.<br>The anti-T7 epitope mouse monoclonal antibody, which specifically recognizes MASMTGGQQMG peptide, was validated by the manufacturer for WB and IP.<br>The anti-p24 monoclonal antibody Nu24 was previously reported and validated for WB, IF, ELISA, and FACS (PMID: 26523972).<br>The anti–β-actin mouse monoclonal antibody, which recognizes an epitope located on the N-terminal end of the β-isoform of actin, was validated by the manufacturer for WB, IF, ELISA, and IHC.

Eukaryotic cell lines

Policy information about cell lines

Cell line source(s)	293T cells (ATCC CRL-3216)<br>HepG2 cells (ATCC HB-806)<br>Human small airway epithelial cells (ScienCell #3230)
Authentication	293T and HepG2 cells were not independently authenticated because they were purchased from ATCC with certificates. Human small airway epithelial cells were isolated by the manufacturer from human lung tissue and cryopreserved at passage one and delivered frozen.
Mycoplasma contamination	Cells were routinely tested negative for mycoplasma contamination (PCR Mycoplasma Detection Set, Takara).
Commonly misidentified lines (See ICLAC register)	No commonly misidentified cell lines were used.

Human research participants

Policy information about studies involving human research participants

Population characteristics	Only deidentified samples were used. Five samples were from confirmed case patients with COVID-19. A negative control was from a healthy donor.
Recruitment	All convalescent patient sera were obtained in February 2020 (before the emergence of the D614G variant) and deidentified at the Self-Defense Forces Central Hospital. A control serum was obtained at the National Institute of Infectious Diseases. All participants provided written informed consent.
Ethics oversight	Experiments using human samples were approved by the Medical Research Ethics Committee of the National Institute of Infectious Diseases, Japan.

Note that full information on the approval of the study protocol must also be provided in the manuscript.
